# Determinants of high platelet reactivity in patients with acute coronary syndromes treated with ticagrelor

**DOI:** 10.1038/s41598-019-40628-0

**Published:** 2019-03-08

**Authors:** Piotr Adamski, Katarzyna Buszko, Joanna Sikora, Piotr Niezgoda, Tomasz Fabiszak, Małgorzata Ostrowska, Malwina Barańska, Aleksandra Karczmarska-Wódzka, Eliano Pio Navarese, Jacek Kubica

**Affiliations:** 1 Collegium Medicum, Nicolaus Copernicus University, Bydgoszcz, Poland; 2 Interventional Cardiology and Cardiovascular Medicine Research, Mater Dei Hospital, Bari, Italy; 3 Mazankowski Alberta Heart Institute, University of Alberta, Edmonton, Alberta Canada

## Abstract

High platelet reactivity (HPR) is a risk factor for stent thrombosis, a potentially lethal complication of percutaneous coronary intervention. HPR is also associated with increased risk of myocardial infarction and death in invasively-treated patients with acute coronary syndrome (ACS). HPR occurs even in ACS patients treated with ticagrelor, a state-of-the-art antiplatelet agent, especially during the first hours of treatment. Patient-level pharmacodynamic data obtained from 102 ACS subjects enrolled in two prospective, pharmacodynamic trials were analysed in order to identify clinical features related with increased odds of on-ticagrelor HPR during the first two hours after ticagrelor loading dose in ACS patients. Presence of ST-segment elevation myocardial infarction (versus non-ST-segment elevation ACS) and morphine co-administration were the strongest predictors of HPR at 1 and 2 hours after ticagrelor loading dose according to linear regression analyses, multiple backward stepwise logistic regression analyses and generalized estimating equation model. By pinpointing simple to recognize clinical features, the results of this study facilitate identification of ACS patients who have the highest odds of HPR during the initial phase of treatment with ticagrelor, and who could potentially benefit from alternative treatment strategies.

## Introduction

Acute coronary syndromes (ACS) constitute a frequent and potentially fatal presentation of coronary artery disease. Excessive activation and aggregation of thrombocytes play a substantial role in the pathophysiology of ACS^1^. Damaged atherosclerotic plaques activate platelets which eventually may result in formation of thrombus obstructing the coronary blood flow and causing myocardial ischaemia^2^. For this reason, limitation of immense platelet activation remains one of the essential therapeutic targets in the treatment of ACS.

Dual antiplatelet therapy, consisting of aspirin and one of P2Y12 receptor inhibitors, is the standard of care in patients with ACS, either treated invasively or conservatively^3,4^. Insufficient platelet inhibition in ACS patients receiving a P2Y12 receptor inhibitor can lead to detrimental consequences, particularly in the setting of percutaneous coronary intervention (PCI). In these patients on-treatment high platelet reactivity (HPR) is an established risk factor for stent thrombosis, a potentially lethal complication^5^. Furthermore, HPR can also be associated with increased risk of myocardial infarction and death^6–8^.

According to the latest European Society of Cardiology (ESC) guidelines ticagrelor is recommended for the treatment of patients with ACS^3,4^. It is a non-thienopyridine, reversible and direct-acting P2Y12 receptor antagonist administered orally^9^. In healthy volunteers and patients with stable coronary artery disease it is quickly absorbed and reaches its maximum plasma concentration within 2 hours after the loading dose (LD), producing expeditious and effective platelet blockade^10^. Moreover, ticagrelor undergoes hepatic biotransformation and has one active metabolite, AR-C124910XX, that exerts similar antiplatelet effect as the parent drug^10,11^. Nevertheless, pharmacokinetics of ticagrelor in ACS patients can be significantly disturbed during the first hours after the LD causing a substantial reduction of its bioavailability and prolongation of time to maximal plasma concentration up to 4–5 hours^12–16^. As a result, failure to reach desired platelet inhibition during the initial hours of ACS therapy is observed in a considerable number of ticagrelor-treated patients^12–14,17^.

One of the main limitations of clopidogrel is its wide interindividual variability in platelet inhibition with a reasonably high number of patients presenting with HPR^18^. Although prasugrel and ticagrelor are characterized by more potent and predictable pharmacodynamics, still as high as 60% of ACS patients may have suboptimal platelet inhibition during the initial hours after the LD^17^. Notably, ACS patients designated to invasive treatment usually receive LD of P2Y12 receptor antagonists within a relatively short period of time before the coronary angiography and PCI.

Recent pharmacodynamic studies have suggested that ACS patients presenting with ST-segment elevation myocardial infarction (STEMI) and those treated with morphine appear to be at increased risk of impaired and postponed platelet blockade following ticagrelor LD^12–15,17,19^. However, little is known about other potential clinical variables influencing acute response to ticagrelor in the setting of ACS.

In this study we sought to identify clinical determinants of HPR at 1 and 2 hours after ticagrelor LD in ACS patients treated invasively.

## Methods

### Study design

A post hoc analysis of combined patient-level pharmacodynamic data from two prospective, phase IV, single centre, investigator-initiated trials (NCT02602444 - observational study; NCT02612116 - randomized, open-label, active-controlled study) was performed to identify clinical characteristics related with impaired antiplatelet effect of ticagrelor in patients with ACS. Both studies were conducted in accordance with the principles contained in the Declaration of Helsinki and Good Clinical Practice guidelines. The trials received a favourable opinion and were approved by The Ethics Committee of Nicolaus Copernicus University in Toruń, Collegium Medicum in Bydgoszcz, Poland (NCT02602444 - approval number KB 617/2015; NCT02612116 - approval number KB 540/2015). All participants of both studies provided a written informed consent prior to any study specific procedures. Study protocols with full lists of inclusion and exclusion criteria, description of methodology, and results were previously published in peer-reviewed journals^12,20–22^.

An individual logistic regression analysis for each of 17 recorded clinical and laboratory variables was planned to identify clinical factors associated with presence of HPR at 1 and 2 hours after a standard 180 mg ticagrelor LD in patients with ACS. The following characteristics were analyzed for each time point: (i) age, (ii) arterial hypertension, (iii) body mass index (BMI), (iv) diabetes mellitus (DM), (v) gender, (vi) glomerular filtration rate (GFR) on admission, (vii) haemoglobin on admission, (viii) history of coronary artery disease, (ix) history of non-haemorrhagic stroke, (x) hyperlipidaemia, (xi) left ventricular ejection fraction <50% at discharge, (xii) mean platelet volume (MPV) on admission, (xiii) morphine administration, (xiv) platelet count on admission, (xv) smoking status, (xvi) type of ACS (STEMI vs. non-ST-segment elevation ACS [NSTE-ACS]), (xvii) formulation of ticagrelor tablets (integral vs. crushed). A multiple stepwise regression analysis, including variables showing impact on the odds of HPR according to our logistic regression analyses, was planned to validate which features are associated with the presence of HPR at 1 and 2 hours after a 180 mg ticagrelor LD.

### Patient population

**Figure 1:**
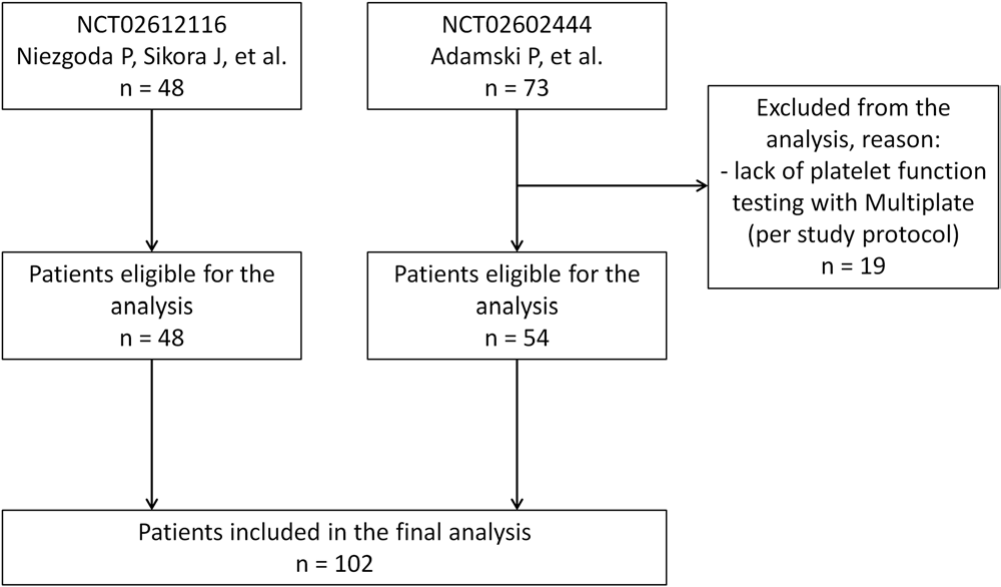
Flow chart of patients included in the analysis.

All subjects included in the current analysis were P2Y12 receptor inhibitor-naive, haemodynamically stable patients qualified for invasive treatment due to ACS (STEMI, non-ST-segment elevation myocardial infarction [NSTEMI] or unstable angina [UA]). The diagnosis of STEMI and NSTEMI was established according to the Third Universal Definition of Myocardial Infarction, and UA was diagnosed according to the 2015 ESC guidelines for the management of NSTE-ACS^3,23^. On top of a 300 mg LD of plain aspirin, all patients received a 180 mg LD of ticagrelor in integral or crushed tablets as a part of dual antiplatelet therapy. During the periprocedural period, all subjects received unfractionated heparin in body weight adjusted dose according to the ESC recommendations. ACS patients enrolled in both studies (n = 121) were screened for availability (Fig. 1), and 102 were eventually included in the current analysis (Table 1). The remaining 19 patients were excluded due to administration of glycoprotein IIb/IIIa inhibitors, which per study protocol precluded full pharmacodynamic assessment in these subjects^12,20^.

**Table 1:** Baseline characteristics of the study participants.

Clinical characteristic	NCT02602444 n = 54	NCT02612116 n = 48	Pooled population n = 102
Age in years	64.1 ± 9.4	64.5 ± 10.2	64.3 ± 9.7
Female	16 (30)	20 (42)	36 (35)
BMI in kg/m^2^	27.0 ± 4.2	28.4 ± 4.9	27.7 ± 4.6
GFR on admission in mL/minute	85.1 ± 15.7	81.6 ± 16.6	83.5 ± 16.1
STEMI	33 (61)	0	33 (32)
NSTE-ACS	21 (39)	48 (100)	69 (68)
NSTEMI	21 (39)	0	21 (21)
UA	0	48 (100)	48 (47)
Crushed ticagrelor tablets	0	32 (67)	32 (31)
Morphine administration	27 (50)	0	27 (26)
History of CAD	8 (15)	27 (56)	35 (34)
Non-haemorrhagic stroke	1 (2)	8 (17)	9 (9)
LVEF at discharge < 50%	32 (59)	17 (35)	49 (48)
Hypertension	28 (52)	39 (81)	67 (66)
Diabetes mellitus	6 (11)	15 (31)	21 (21)
Hyperlipidaemia	49 (91)	41 (85)	90 (89)
Current smoker	23 (43)	11 (23)	34 (33)
Chronic obstructive pulmonary disease	0	2 (2)	2 (2)
Peripheral artery disease	4 (7)	0	4 (4)
Gout	2 (4)	0	2 (2)
Platelets on admission in 10^9^/L	228 ± 60	220 ± 54	224 ± 57
Mean platelet volume on admission in fL	10.9 ± 1.0	10.6 ± 0.9	10.8 ± 1.0
Haemoglobin on admission in g/dL	14.4 ± 1.5	14.2 ± 1.3	14.3 ± 1.4

Data are shown as mean ± standard deviation or number (%). BMI: body mass index, CAD: coronary artery disease, GFR: glomerular filtration rate, LVEF: left ventricular ejection fraction, NSTE-ACS: non-ST-elevation acute coronary syndrome, NSTEMI: non-ST-elevation myocardial infarction, STEMI: ST-elevation myocardial infarction, UA: unstable angina.

### Concomitant treatment

All patients in both studies were treated according to the ESC guidelines, and received beta-blockers, statins, and angiotensin-converting enzyme inhibitors or angiotensin II receptor blockers, if not contraindicated. Morphine was used at the discretion of the attending physician. The type of implanted stent and choice of the access site for coronary invasive procedure (radial or femoral) was at the discretion of the operator. Administration of glycoprotein IIb/IIIa receptor inhibitors was restricted only to bailout situations.

### Blood sampling

Blood samples for evaluation of platelet reactivity were obtained from an 18-gauge venous cannula inserted into one of the forearm veins. The first 5 mL portion of blood was discarded to avoid spontaneous platelet activation. Blood samples were collected at pre-defined time points according to the study protocols^20,21^, however the current analysis was restricted to pharmacodynamic data acquired from samples drawn before ticagrelor LD, 1 hour and 2 hours after ticagrelor LD. Baseline blood samples were obtained before the administration of unfractionated heparin.

### Pharmacodynamic assessment

Platelet function testing was performed using Multiplate analyzer (Roche Diagnostics International Ltd., Rotkreuz, Switzerland) according to the manufacturer’s instructions^24^. The pharmacodynamic evaluation was performed for each sample within 1 hour of collection. Patients requiring administration of glycoprotein IIb/IIIa receptor inhibitors were excluded from platelet testing with Multiplate, as it could have had influenced the results. In line with the available literature, HPR was defined as platelet reactivity >46 units (U)^5,6^.

### Statistical analysis

Statistical calculations were performed using the Statistica 13 package (StatSoft, Tulsa, OK, USA), Matlab 2017b (MathWorks, Natick, MA, USA) and R version 3.5.0 (The R Foundation, Vienna, Austria). Data for age, BMI, GFR, haemoglobin concentration, MPV, platelet count and platelet reactivity were presented as means with standard deviations. The pharmacodynamic continuous data were transformed to dichotomous variables according to the threshold of HPR. As the study population was not numerous enough to perform a reliable multiple regression analysis for all of the collected variables at once, first an individual logistic regression analysis for each of the analyzed characteristics was performed to identify features independently associated with platelet reactivity above the HPR threshold at 1 and 2 hours after ticagrelor LD. Next, a multiple backward stepwise logistic regression analysis, including clinical features that were indicated as independently associated with presence of HPR in the logistic regression analyses, was performed for both time points to validate the previous findings. Variables were removed from further steps of multiple backward stepwise logistic regression analyses in case of p-value > 0.05. Finally, we wanted to analyze the dynamics of HPR changes with respect to the pharmacological treatment and modeling factors. To interpret these discrete longitudinal data where the examined effect is affected by certain correlation of factors within the subjects, we applied a generalized estimating equation (GEE) model dedicated for analysis of dichotomous longitudinal data with repeated measures in the same group of patients over time^25^. In the GEE model we assumed binominal data distribution and independent correlation structure. In all cases, p-values < 0.05 were considered significant.

## Results

In a logistic regression analysis STEMI versus NSTE-ACS (odds ratio [OR] 10.55, 95% confidence interval [CI] 4.00–27.80, p = 0.00001), co-administration of morphine (OR 13.63, 95% CI 4.78–38.86, p = 0.000001), administration of integral versus crushed ticagrelor tablets (OR 26.11, 95% CI 3.37–201.99, p = 0.002) and platelet count on admission (OR 1.01, 95% CI 1.00–1.02, p = 0.025) were found to be independent predictors of HPR at 1 hour after administration of 180 mg ticagrelor LD (Table 2). At 2 hours after ticagrelor LD solely presence of STEMI (OR 9.29, 95% CI 2.70–31.89, p = 0.0004), morphine use (OR 9.63, 95% CI 2.93–31.59, p = 0.0002) and platelet count on admission (OR 1.01, 95% CI 1.00–1.02, p = 0.028) remained associated with increased odds of HPR (Table 2). Patients with a history of coronary artery disease had lower odds of HPR at 1 hour (OR 0.23, 95% CI 0.08–0.67, p = 0.007), while history of arterial hypertension was related with reduced odds of HPR both at 1 and 2 hours following ticagrelor LD (1 hour: OR 0.33, 95% CI 0.14–0.79, p = 0.013; 2 hours: OR 0.25, 95% CI 0.08–0.75, p = 0.013).

**Table 2:** Influence of clinical characteristics on the odds of high platelet reactivity at 1 and 2 hours after ticagrelor loading dose according to the linear regression analyses.

Clinical characteristic	1 hour after ticagrelor LD			2 hours after ticagrelor LD		
	Odds ratio for HPR	95% confidence interval	p value	Odds ratio for HPR	95% confidence interval	p value
Age (per year)	1.03	0.99–1.08	0.15	0.99	0.94–1.05	0.85
Female	2.13	0.84–5.41	0.11	2.70	0.71–10.19	0.14
BMI (per kg/m2)	1.03	0.94–1.12	0.58	n/a	n/a	n/a
GFR at admission (per mL/minute)	1.01	0.98–1.04	0.46	0.98	0.95––1.01	0.25
STEMI (vs. NSTE-ACS)	10.55	4.00–27.80	0.00001	9.29	2.70–31.89	0.0004
Integral ticagrelor tablets (vs. crushed ticagrelor tablets)	26.11	3.37–201.99	0.002	n/a	n/a	n/a
Morphine administration	13.63	4.78–38.86	0.000001	9.63	2.93–31.59	0.0002
History of CAD	0.23	0.08–0.67	0.007	0.39	0.10–1.47	0.16
Nonhaemorrhagic stroke	0.25	0.03–2.06	0.20	n/a	n/a	n/a
LVEF at discharge < 50%	1.17	0.50–2.70	0.72	1.79	0.60–5.36	0.30
Hypertension	0.33	0.14–0.79	0.013	0.25	0.08–0.75	0.013
Diabetes mellitus	1.41	0.54–3.68	0.48	0.63	0.20–2.05	0.45
Hyperlipidaemia	0.63	0.18–2.17	0.47	0.31	0.08–1.18	0.09
Current smoker	1.81	0.76–4.28	0.18	1.24	0.41–3.76	0.70
Platelets on admission (per 10^9^/L)	1.01	1.00–1.02	0.025	1.01	1.00–1.02	0.028
Mean platelet volume on admission (per fL)	1.23	0.79–1.91	0.35	1.53	0.87–2.70	0.14
Haemoglobin on admission (per g/dL)	1.07	0.79–1.44	0.67	1.17	0.80–1.72	0.40

BMI: body mass index, CAD: coronary artery disease, GFR: glomerular filtration rate, HPR: high platelet reactivity, LD: loading dose, LVEF: left ventricle ejection fraction, n/a: not available due to the lack of unique solutions for the equations of the model, NSTE-ACS: non-ST-elevation acute coronary syndrome, STEMI: ST-elevation myocardial infarction.

A backward stepwise multiple regression analysis validated that diagnosis of STEMI (OR 6.26, 95% CI 2.15–18.27, p = 0.0008) and co-administration of intravenous morphine (OR 8.17, 95% CI 2.62–25.46, p = 0.0003) are related with increased odds of HPR at 1 hour after ticagrelor LD, while no relation was revealed at this time point regarding the type of ticagrelor tablets, history of coronary artery disease, arterial hypertension or platelet count (Table 3). Accordingly, presence of STEMI (OR 5.04, 95% CI 1.32–19.32, p = 0.018) and use of morphine (OR 5.20, 95% CI 1.43–18.99, p = 0.013) were also related with higher odds of HPR at 2 hours following ticagrelor LD. Again presence of hypertension and platelet count had no significant impact on the prevalence of HPR (Table 3).

**Table 3:** Results of backward stepwise multiple regression analyses evaluating influence of clinical characteristics on the odds of high platelet reactivity at 1 and 2 hours after ticagrelor loading dose.

Clinical characteristic	1 hour after ticagrelor LD			2 hours after ticagrelor LD		
	Odds ratio for HPR	95% confidence interval	p value	Odds ratio for HPR	95% confidence interval	p value
STEMI (vs. NSTE-ACS)	6.26	2.15–18.27	0.0008	5.04	1.32–19.32	0.018
Morphine administration	8.17	2.62–25.46	0.0003	5.20	1.43–18.99	0.013
Hypertension	n/a	n/a	>0.05	n/a	n/a	>0.05
Integral ticagrelor tablets (vs. crushed ticagrelor tablets)	n/a	n/a	>0.05	—	—	—
History of CAD	n/a	n/a	>0.05	—	—	—
Platelets on admission (per 10^9^/L)	n/a	n/a	>0.05	n/a	n/a	>0.05

These analyses were performed only for clinical variables that were indicated as independently associated with presence of HPR in the logistic regression analyses (Table 2). The above table depicts the results of the last step of multiple backward stepwise logistic regression analyses. Variables were removed from each further step of multiple backward stepwise logistic regression analysis in case of p value > 0.05, therefore no OR or CI are available for these characteristics. CAD: coronary artery disease, dash sign (−): variable not included in analysis as it has not been pinpointed as independently associated with HPR in certain time point in linear regression analysis, HPR: high platelet reactivity, LD: loading dose, n/a: not available, NSTE-ACS: non-ST-segment elevation acute coronary syndrome, STEMI: ST-segment elevation myocardial infarction.

The GEE model validated findings from the previous analyses showing that both STEMI (OR 4.99, 95% CI 1.88–13.20, p = 0.001) and morphine use (OR 5.67, 95% CI 1.90–16.94, p = 0.002) were linked to increased odds of HPR during the first 2 hours after ticagrelor LD. Additionally, the GEE model has shown that odds of HPR increase with the magnitude of baseline platelet reactivity evaluated before ticagrelor LD (OR 1.04, 95% CI 1.02–1.06, p < 0.0001) and decrease with the passing time between 1 and 2 hours after ticagrelor LD (OR 0.17, 95% CI 0.06–0.50, p = 0.001). Detailed results of the GEE model are presented in Table 4.

**Table 4:** The results of generalized estimating equation model evaluating high platelet reactivity predictors.

Clinical characteristic	Odds ratio for HPR	95% confidence interval	Estimate	Robust SE	p value
Intercept	0.01	0.00–0.05	−4.97	1.05	<0.0001
Time lapse from 1 to 2 h after ticagrelor LD	0.17	0.06–0.50	−1.75	0.54	0.001
STEMI (vs. NSTE-ACS)	4.99	1.88–13.20	1.60	0.50	0.001
Morphine administration	5.67	1.90–16.94	1.74	0.56	0.002
Baseline platelet reactivity (per Multiplate unit)	1.04	1.02–1.06	0.04	0.01	<0.0001
Platelets on admission (per 10^9^/L)	1.00	0.99–1.01	−0.01	0.01	0.65

h: hours, HPR: high platelet reactivity, LD: loading dose, NSTE-ACS: non-ST-segment elevation acute coronary syndrome, SE: standard error, STEMI: ST-segment elevation myocardial infarction.

The prevalence of HPR among patients with STEMI was 67% and 36% at 1 and 2 hours, respectively. The occurrence of HPR in patients treated with morphine was 74% and 41% at the same time points.

## Discussion

HPR is a perilous phenomenon that can negatively affect clinical outcomes and increase the risk of thrombotic complications in patients with ACS receiving PCI^5–8^. Pharmacodynamic data from available trials clearly show that the prevalence of HPR in patients with ACS is highest during the first hours after administration of ticagrelor LD^12–14,17^. Numerous ACS patients receive P2Y12 receptor inhibitor LD shortly before the coronary angiography or PCI. Therefore, ACS patients treated invasively are at substantial risk of insufficient platelet inhibition directly after stent implantation, even if they receive a state-of-the-art antiplatelet agent, such as ticagrelor. Consequently, we sought to identify clinical characteristics related with increased risk of HPR during the first two hours after ticagrelor LD.

The current study represents an analysis of combined, patient-level platelet reactivity data obtained from 102 participants of two prospective trials performed at our centre^12,22^. Importantly, the investigated data represent a wide spectrum of ACS patients designated to invasive treatment, including subjects with STEMI, NSTEMI and UA.

The analyses performed for time points 1 and 2 hours after the intake of ticagrelor LD revealed two clinical characteristics that were consistently and strongly related with increased odds of HPR. In our study, patients presenting with STEMI or receiving intravenous morphine had 5-fold to 8-fold higher odds of HPR at 1 and 2 hours after ticagrelor LD, than individuals with NSTE-ACS or opioid-naive patients, respectively. Taking into account the fact that even 24–42% of NSTE-ACS and 28–55% of STEMI patients may receive morphine to alleviate chest pain, the population of ACS patients at risk of HPR during the acute phase of treatment is significant^12,26,27^.

Our findings are in line with the results of pharmacodynamic studies showing a significant delay in the onset of platelet inhibition obtained with ticagrelor LD in ACS patients treated with morphine or presenting with STEMI^12–15,17,19^. STEMI as well as use of morphine result in approximately 35% reduction in total ticagrelor bioavailability during the first hours after the LD, which leads to postponed and diminished platelet inhibition in the initial phase of treatment^12,13^. The impaired pharmacodynamics of ticagrelor observed in these patients most likely result from lagged and reduced absorption of the drug due to decreased propulsive motility of the gastrointestinal tract present in STEMI and morphine-treated ACS subjects^12,28^. This issue has been acknowledged in the latest ESC guidelines on the treatment of STEMI, where the class of recommendation for analgesia with morphine in this setting has been lowered from I to IIa, with a level of evidence C^4,29^.

Generally, our GEE model has underlined that time lapse from LD is crucial for the ticagrelor antiplatelet effect onset. In our analysis passage of 60 minutes between 1 and 2 hours after ticagrelor LD was related with 83% reduction of HPR odds. Again, presence of STEMI and morphine administration have been pinpointed as factors strongly increasing the odds of HPR early after ticagrelor LD administration. Moreover, we showed that the higher the baseline platelet reactivity before ticagrelor LD, the higher the odds of HPR.

So far, several approaches to improve the pharmacokinetics and pharmacodynamics of ticagrelor in ACS have been evaluated. Ticagrelor tablets crushing accelerates the absorption and subsequently the onset of antiplatelet action in STEMI patients^30,31^. In line with these data, administration of integral versus crushed ticagrelor tablets was an independent predictor of HPR at 1 hour post-LD in our logistic regression analysis. Alternative method of ticagrelor administration are chewed tablets. This formulation provides a quicker onset of platelet inhibition than integral pills during the initial 60 minutes after ticagrelor LD in both STEMI and NSTEMI patients^32,33^. Moreover, different analgesic strategies to alleviate ischaemia-driven chest pain were proposed to avoid or surpass the negative impact of morphine on ticagrelor bioavailability and its antiplatelet action^34^. Currently ongoing studies are expected to evaluate the feasibility, efficacy and safety of equimolar oxygen and nitrous oxide mixture in combination with paracetamol as a replacement for morphine (NCT02198378), addition of metoclopramide to improve the gastrointestinal passage and absorption of ticagrelor (NCT02939235, NCT02627950), use of oral naloxone to counteract the peripheral action of morphine (NCT02939248), and use of subcutaneous, peripherally acting mu-opioid receptor antagonist methylnaltrexone (NCT02403830). On the other hand, fentanyl probably should not be considered as a superior alternative for morphine in patients receiving PCI. Results of the PACIFY study show that administration of intravenous fentanyl in invasively-treated patients causes decreased bioavailability of ticagrelor and higher prevalence of HPR at 2 hours after ticagrelor LD, as seen with morphine injection^35,36^. Finally, from a pharmacodynamic point of view, ACS patients with increased odds of HPR during the first hours after ticagrelor LD could benefit from cangrelor, the only available parenteral P2Y12 receptor antagonist. Infusion of cangrelor provides almost instant, but also a powerful and reversible platelet inhibition^37^. It has to be noted though, that currently cangrelor may be considered only in STEMI and NSTE-ACS patients not pre-treated with oral P2Y12 receptor inhibitors^3,4^. Thus, proper identification of ACS patients at risk of early on-ticagrelor HPR might be crucial for the correct selection of subjects who could particularly benefit from cangrelor.

Platelet count after being indicated as potentially affecting the HPR odds in the linear regression analyses was found not to significantly affect the odds for HPR in further analyses. Interestingly, our logistic regression models indicated arterial hypertension and history of coronary artery disease as conditions related with reduced odds ratio of HPR in the early phase of ACS treatment with ticagrelor. In the current study, patients with a history of hypertension had 66% and 75% lower odds of having platelet reactivity above the threshold for HPR at 1 and 2 hours after ticagrelor LD, respectively. Alike, prior diagnosis of ischaemic heart diseases was associated with 77% lower odds of HPR at 1 hour. Of note, none of these features was further validated in the multiple stepwise backward regression analyses. To our knowledge, this is the first report suggesting a beneficial effect of hypertension and previous coronary artery disease on the pharmacodynamic efficacy of ticagrelor in ACS patients. Nevertheless, it has to be considered that this observation might have been just a play of chance as it has not been confirmed in subsequent analyses, and the potential mechanism behind this finding is obscure and uncertain.

### Study limitations

Several restraints resulted from the design of the analyzed studies. Neither trial was intended to evaluate clinical endpoints nor included enough patients to perform such assessment. Therefore, we were unable to evaluate the relationship of our findings with adverse clinical events. Data on time from chest pain to ticagrelor LD or to PCI, and time relationship between administration of morphine and ticagrelor LD were not fully recorded and for this reason were not included in the analyses. Next, smoking status was collected solely based on a statement made by each patient, and was not validated objectively. Admittedly our results on impact of tablets crushing on odds of HPR might have been affected by the fact that all patients with myocardial infarction received integral ticagrelor tablets. As mentioned earlier, the study population was not numerous enough to include all of the collected variables in one multiple regression analysis model. Thus, multiple regression analyses included only clinical variables that were shown to be independent predictors of HPR in linear regression analyses. Finally, only one method of platelet function assessment was used in the current analysis. Nevertheless, it has to be mentioned that Multiplate is one of three currently recommended assays to evaluate platelet inhibition, and that correlation between ticagrelor concentrations and platelet reactivity evaluated with Multiplate is comparable with two remaining assays^6,15^.

## Conclusions

Presence of STEMI and morphine co-administration are the strongest predictors of HPR at 1 and 2 hours after ticagrelor LD in patients with ACS.

## Data Availability

The dataset from NCT02602444 trial analyzed during the current study is available in the figshare.com repository (doi:10.6084/m9.figshare.5396989). The dataset from NCT02612116 trial analyzed during the current study are available from the corresponding author on reasonable request.
